# Deep sequencing of uveal melanoma identifies a recurrent mutation in *PLCB4*

**DOI:** 10.18632/oncotarget.6614

**Published:** 2015-12-14

**Authors:** Peter Johansson, Lauren G. Aoude, Karin Wadt, William J. Glasson, Sunil K. Warrier, Alex W. Hewitt, Jens Folke Kiilgaard, Steffen Heegaard, Tim Isaacs, Maria Franchina, Christian Ingvar, Tersia Vermeulen, Kevin J. Whitehead, Christopher W. Schmidt, Jane M. Palmer, Judith Symmons, Anne-Marie Gerdes, Göran Jönsson, Nicholas K. Hayward

**Affiliations:** ^1^ QIMR Berghofer Medical Research Institute, Brisbane, QLD, Australia; ^2^ Department of Clinical Genetics, Rigshospitalet, Copenhagen, Denmark; ^3^ The Terrace Eye Centre, Brisbane, QLD, Australia; ^4^ Menzies Institute for Medical Research, University of Tasmania, Hobart, TAS, Australia; ^5^ Lions Eye Institute, University of Western Australia, Perth, WA, Australia; ^6^ Department of Ophthalmology, Rigshospitalet-Glostrup Hospital, University of Copenhagen, Copenhagen, Denmark; ^7^ Department of Pathology, Rigshospitalet, University of Copenhagen, Copenhagen, Denmark; ^8^ Department of Clinical Sciences, Lund University, Lund, Sweden; ^9^ The Royal Perth Hospital, Perth, WA, Australia; ^10^ Sullivan Nicolaides Pathology, Brisbane, QLD, Australia

**Keywords:** uveal melanoma, recurrent mutation, PLCB4, copy number variation, structural variants

## Abstract

Next generation sequencing of uveal melanoma (UM) samples has identified a number of recurrent oncogenic or loss-of-function mutations in key driver genes including: *GNAQ*, *GNA11*, *EIF1AX*, *SF3B1* and *BAP1*. To search for additional driver mutations in this tumor type we carried out whole-genome or whole-exome sequencing of 28 tumors or primary cell lines. These samples have a low mutation burden, with a mean of 10.6 protein changing mutations per sample (range 0 to 53). As expected for these sun-shielded melanomas the mutation spectrum was not consistent with an ultraviolet radiation signature, instead, a BRCA mutation signature predominated. In addition to mutations in the known UM driver genes, we found a recurrent mutation in *PLCB4* (c.G1888T, p.D630Y, NM_000933), which was validated using Sanger sequencing. The identical mutation was also found in published UM sequence data (1 of 56 tumors), supporting its role as a novel driver mutation in UM. PLCB4 p.D630Y mutations are mutually exclusive with mutations in *GNA11* and *GNAQ*, consistent with PLCB4 being the canonical downstream target of the former gene products. Taken together these data suggest that the *PLCB4* hotspot mutation is similarly a gain-of-function mutation leading to activation of the same signaling pathway, promoting UM tumorigenesis.

## INTRODUCTION

Uveal melanoma (UM) is the most common primary intraocular malignancy in adults with an incidence of 5.6 cases per million per year between 1973 and 2008 reported in the United States [1]. The number of newly reported cases of UM over this time frame remained at a relatively steady rate, suggesting that the influence of ultraviolet radiation on UM is considerably smaller than the association observed with cutaneous melanoma (CMM).

In UM a recurrent somatic mutation occurs frequently in *GNAQ*, encoding an alpha subunit of heterotrimeric G proteins, located at chromosome band 9q21 [2]. *GNA11*, a paralog of *GNAQ* located in chromosome band 19p13.3, is also recurrently mutated at the same codon [3]. Hotspot GNAQ p.Q209 mutations are found in 45% of primary UM and 22% of metastases, while GNA11 p.Q209 mutations are found in 32% of primary tumors and 57% of UM metastases. A second mutation hotspot has also been identified at codon p.R183 in both genes. Overall, 83% of UM acquire mutations in either *GNAQ* or *GNA11*. Unlike some other somatic mutations in UM these are not associated with prognosis.

There are also commonly occurring loss-of-function mutations in the tumor suppressor gene *BAP1* (BRCA1 associated protein-1) located on chromosome 3 [4]. These mutations are associated with worse prognosis. Approximately 40% of UM harbor inactivating somatic mutations in *BAP1*, which occur along the length of the gene and generally result in protein truncations. Metastasizing tumors are also associated with monosomy 3, suggesting loss of both copies of *BAP1* is necessary [5]. There are also commonly occurring gene expression profiles whereby metastatic tumors present with clusters of up-regulated genes on chromosome 8q, and down-regulated genes on chromosomes 3 and 6q [6, 7].

There are two further frequently mutated genes in UM. Recurrent mutations in *SF3B1* occur at codon 625 in approximately 18% of tumors and are associated with better prognosis [8], as are mutations in *EIF1AX* [9].

## RESULTS AND DISCUSSION

### Protein altering single nucleotide variants and small insertion/deletions

We performed WGS or WES on 28 untreated UM samples and identified a total of 297 non-synonymous mutations (mean 10.6, range 0 to 53, Supplementary Table 1), including both single nucleotide variants (SNVs) and indels (Supplementary Table 2).

We first assessed mutations in known UM drivers and detected 11 mutations in *BAP1* (7 frameshifting indels, 2 splice mutations, 1 nonsense mutation and 1 missense mutation), 14 mutations occurred at GNA11 p.Q209P, 7 mutations occurred at GNAQ p.Q209P and a single mutation at GNAQ p.G48L. As expected the mutations in GNA11 and GNAQ were mutually exclusive. We detected 4 mutations occurring in EIF1AX (p.P2L, p.G6V, p.G8R and a splice mutation) which were found to be mutually exclusive with *BAP1* mutations. We also detected 3 mutations in SFB31: p.R625C, p.R625H and p.K666T (Table 1, Supplementary Table 2).

**Table 1:** Driver mutations in UM

Tumor ID	BAP1	EIF1AX	GNA11	GNAQ	PLCB4	SF3B1
00028-001-CL	p.D73Vfs*4		p.Q209L			p.R625C
00038-001-CL			p.Q209L			
00061-001-CL	p.R699Qfs*6		p.Q209L			
00085-001-CL	p.H224Qfs*14		p.Q209L			
00099-001-CL			p.Q209L			
141378RG-T						p.R625H
258	p.F170Lfs*13				p.D630Y	p.K666T
531		p.G8R	p.Q209L			
533	p.G579Efs*63		p.Q209L			
534			p.Q209L			
535		splice		p.Q209P		
537			p.Q209L			
538		p.G6V	p.Q209L			
539			p.Q209L			
550	p.V346Sfs*51			p.Q209P		
552	p.G41_54del			p.G48L		
553			p.Q209L			
554						
556	Splice			p.Q209P		
557		p.P2L		p.Q209P		
C0622943-T			p.Q209L			
ETB-0002-T	Splice			p.Q209P		
J2217960PR-T				p.Q209P		
K0111890AC-T				p.Q209P		
MM1488-T						
MM1551-T	p.Q684X				p.D630Y	
MM1563-T						
MM639-T	p.G128R		p.Q209L			

To identify novel UM driver genes we searched for additional recurrent mutations. *PLCB4*, phospholipase C, beta 4, was the only other gene that had a recurrent mutation (c.G1888T, p.D630Y, chr20:9389753, NM_000933), which occurred in 2 of 28 samples. This mutation is predicted to be functionally deleterious by SIFT (http://string-db.org/ and probably damaging by PolyPhen2. It is also at a highly conserved site across species (Supplementary Figure 1) with a GERP++ score of 5.69. This mutation is located in the Y-domain of the highly conserved catalytic core of PLCB4, which plays a vital role in the intracellular transduction of extracellular signals in the retina. Notably, this protein is a downstream target of GNAQ/GNA11 and is activated by direct interaction with GNAQ [10]. A STRING interaction network (http://string-db.org/) shows direct binding of PLCB4 to GNAQ and GNA11 (Supplementary Figure 2). The PLCB4 interactome also shows binding of PLCB3 to GNAQ and GNA11. Interestingly, in addition to the hotspot PLCB4 mutation, 1 of 28 UM samples had a novel mutation in *PLCB3*, phospholipase C beta 3 (c.G2694C, p.K898N, chr11:64032834, NM_001184883). The location of this mutation is within the CTD linker which plays a significant role in GNAQ activation [10]. The two samples we identified with PLCB4 mutations did not have mutations in either GNAQ or GNA11. A search of mutations in other UM WGS/WES data sets [8, 9, 11, 12] identified the same PLCB4 mutation in 1 of 56 samples, which also occurred mutually exclusive to GNAQ and GNA11 mutations. Taken together these data suggest that the PLCB4 hotspot mutation is similarly a gain-of-function mutation leading to activation of the same signaling pathway. Of note, *PLCB4* is highly mutated (21% to 28%) in CMM [11, 13-15] but in contrast to UM, these mutations occur along the length of the gene, suggestive of them being loss-of-function. Consistent with this notion, none of the 159 reported non-synonymous *PLCB4* mutations in CMM occur at the recurrent hotspot we observe in UM. While, speculative, this suggests context specific roles where *PLCB4* is an oncogene in UM and a tumor suppressor in CMM.

Other than *PLCB4* and the previously characterized UM driver genes, only 4 additional genes were mutated in more than one sample (Supplementary Table 2). Two missense mutations were found in each of *MUC3A, TCHH*, *TTN* and *LLGL1*. Only the latter has previously been associated with cancer, being a tumor suppressor in glioblastoma and oesophageal squamous cell carcinoma [16, 17], aberrantly spliced in hepatocellular carcinoma [18] and with reduced expression contributing to disease progression in CMM [19]. This makes it a plausible candidate driver gene for UM but further studies are required to determine its potential contribution to the development of this cancer type.

### Genomic landscape

### Mutation burden

The 14 samples on which we performed WGS allowed a more global assessment of the genomic landscape of UM. Firstly, to assess the mutation burden we calculated the number of mutations per megabase (Mb). The mean mutation rate across the UM genomes was 0.50/Mb (range 0.22-0.66/Mb) and the mutation load was similar in protein coding regions (mean 0.53, range 0.06-2.52/Mb, Supplementary Table 1).

### Mutation signatures

**Figure 1:**
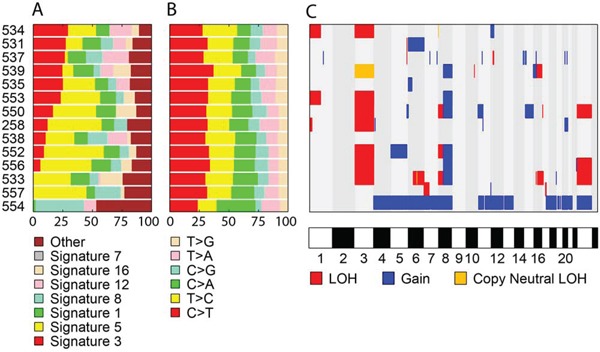
Genomic landscape of UM **A.** Each color represents a known mutation signature as defined by Alexandrov *et al.* [20]. **B.** Proportion of base changes observed in each sample. **C.** Summary of chromosomal aberrations.

To explore the underlying mutational processes in UM, we compared the mutational spectra with the signatures previously identified by Alexandrov *et al.* [20]. As expected, most samples showed signatures 1 and 5, which occur ubiquitously in all cancer types (Figure 1). We also noticed significant contributions of signatures 12 or 16 in almost every sample. The majority (79%) of samples also had signature 3, which is associated with defects in DNA double-strand break-repair (the ‘BRCA’ signature) but there is no significant association between BAP1 mutation and this signature in the samples analyzed here. Notably, no sample showed signature 7, which is frequently observed in CMM and associated with ultraviolet light exposure.

### Copy number aberrations

Chromosomal segments showing aberrant copy number variation (CNV) or loss of heterozygosity (LOH) are shown in Figure 1. The most common event was monosomy 3 or copy neutral LOH of the entire chromosome 3, which was seen in 8 of 14 samples (57%). Notably, all samples with *BAP1* mutation (n=6) were hemizygous for chromosome 3. Other frequent aberrations included 8q gain (n=6), 8p loss (n=3), 16q loss (n=2), and X loss (n=3). These common CNVs were only observed in samples with monosomy 3 (n=7) or copy neutral chromosome 3 LOH (n=1), whereas samples with diploid chromosome 3 tended to have fewer large chromosomal aberrations. The chromosomal profile of sample 554 looked quite different compared to the others and had gained copies of almost every chromosome; specifically triploid chromosomes 4, 5, 6, 11, 12, 13, 17q, and 21, and tetraploid chromosomes 7, 8, 18, 20, and X.

### Chromosome 8q duplication is a late event

**Figure 2:**
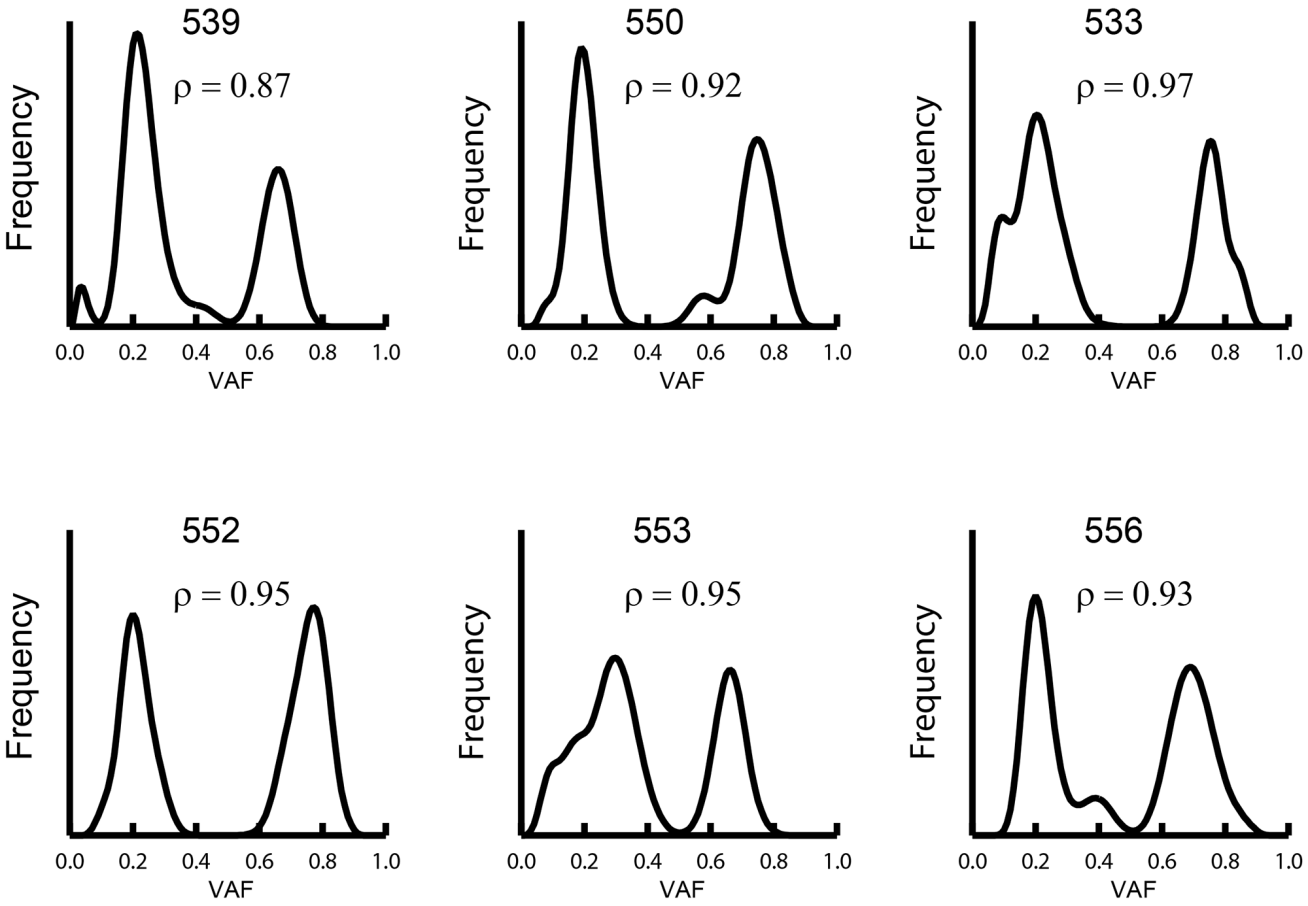
Distribution of somatic VAF on chromosome 8q in samples with gained copies of chromosome 8q The fraction of mutations, ρ, occurring prior chromosomal gain was estimated using a mix-model (see Methods). The curve was generated using a Bayesian smoother in which the likelihood for the data was calculated for each mutation and the sum of these likelihoods was used as the estimate of the VAF distribution.

We observed six tumors with gained chromosome 8q. To assess whether this aberration occurred early or late in the tumor development, we examined the variant allele frequency (VAF) for the somatic mutations on chromosome 8q. If the duplication is an early event, somatic mutations are only observed in one of the copies, whereas if the duplication occurs later, half the mutations are duplicated and the VAF distribution is bimodal. Using this observation we examined the six samples with gained chromosome 8q, and as seen in Figure 2 the VAF distribution in these samples was distinctly bimodal. On average 93% (range 87-97%) of the mutations were estimated to occur before the duplication showing that gained chromosome 8q is a late event.

### Structural variants

**Figure 3:**
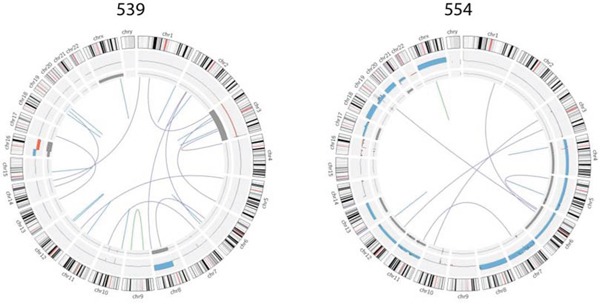
Summary of large genomic variations in two typical samples Structural variants are illustrated by an edge between its two breakpoints, where the color describes the type of variant including interchromosomal (purple), deletion (blue), translocation/tandem repeat (red), and inversion (green). The middle layer illustrates allelic imbalance and LOH; the width of the segments describes how much the minor allele frequency of the segment deviate from normal (0.5). The outer layer illustrates chromosomal loss (red) and gain (blue).

We detected a total of 297 SVs in the 14 samples subjected to whole-genome sequencing (mean 273, range 9 to 42, Supplementary Tables 3 and 4). These consisted of large deletions, gene fusions, regions of tandem duplication and truncated genes. Of these, there were 7 genes that were affected more than once (*CDH13*, *FAM135B*, *GFRAL*, *LRRC16A*, *MOK*, *SEMA3E* and *VPS13B*). We observed only two in-frame fusion events. Sample 553 has a fusion between *GSPT1* and *HSD17B3* and 550 has a fusion between *FAM135B* and *PDSS2* (Figure 3). The latter occurs between chromosomes 6 and 8 which are two of the most frequently aberrant chromosomes in UM. In sample 539 we observed an intronic deletion of unknown consequence encompassing 154bp of *BAP1*. Interestingly, 539 has copy-neutral LOH of chromosome 3.

## CONCLUSION

Next generation sequencing of 28 UM samples shows that overall these tumors have a low SNV and SV mutation burden. The majority of tumors presented with a BRCA mutation signature, and as expected these sun-shielded melanomas had no ultraviolet radiation signature. Analysis of SNVs identified a novel recurrent mutation in PLCB4 (p.D630Y). The identical mutation is also seen in 1 of 56 UM tumors with published sequence data. PLCB4 is the canonical downstream target of GNAQ/GNA11 and is activated by direct interaction with GNAQ. This novel mutation is a likely driver in UM and occurs mutually exclusively with *GNAQ/GNA11* mutations. Taken together these data suggest that the *PLCB4* hotspot mutation is a gain-of-function mutation leading to activation of the same signaling pathway. Functional studies are warranted to further characterize the role of *PLCB4* in UM.

## MATERIALS AND METHODS

### Ethics

Ethics approvals were obtained from the Human Research Ethics Committees of the QIMR Berghofer Medical Research Institute, the University of Western Australia, the Capital Region of Denmark, and Lund University. Written informed consent was obtained from each participant in this study.

### Next generation sequencing

Whole-genome sequencing (WGS) was performed on 14 primary UM tumors and whole-exome sequencing (WES) was performed on 9 UM tumors and 5 cell lines established from primary UM tumors. Supplementary Table 5 details the histology of the samples, which were sequenced along with matching germline samples extracted from blood or saliva.

Sequence reads were aligned against the human reference genome (build 19) using BWA [21], duplicate reads were marked with Picard, and reads were realigned against known insertion/deletions (indels) and base qualities were recalibrated using GATK [22]. Somatic mutations and short indels were identified using Samtools/bcftools [23] and further annotated using ANNOVAR [24]. A stringent set of filtering criteria was applied to the WGS/WES output to reduce the false positive rate. Firstly the germline samples were filtered by eliminating all population variants reported in dbSNP (unless also reported in COSMIC). The germline variants were further filtered so only variants with no reads for the variant allele and ≥ 8 reads for the reference allele were included. The variants in the tumor were filtered so that only those with Phred-scaled genotype likelihood (PL) for wildtype ≥ 60 and ≥ 4 variant reads were included. Furthermore, any variants with quality warnings regarding regions of duplication, tri-allelic variants, and variants located at the tail end of reads were eliminated. Lastly the constrained log likelihood ratio (CLR) score was required to be ≥ 40; the higher the score the more likely that a variant is truly somatic. Non-frameshift variants that passed this set of filtering criteria were hand-curated, whereby variants in regions of trinucleotide expansions or reductions were removed from the dataset as they are likely due to poor mapping.

Structural variants (SV) were identified using Janda v0.8.1 (http://janda.sourceforge.net/). Janda uses both discordant read pairs and clipped reads to detect breakpoints. These were then filtered using the following criteria: SVs were required to be supported by at least three junction reads; the junction reads aligned against the junction with at most two mismatches on average. Breakpoints with extremely high coverage (>1000x) in either sample were discarded as well as breakpoints with low coverage (<2x) in the normal sample. To avoid artifacts due to segmental duplication and erroneous mapping, we removed breakpoints that overlapped with the Genomic Super Dups Database (http://humanparalogy.gs.washington.edu/SDD), or if the 20bp regions around the two breakpoints were too similar (≥90%), as well as breakpoints that were detected in multiple samples. Lastly, if the junction contained an insertion greater than 3bp inserted between the breakpoints, the variant was removed.

Copy number aberrations and/or allelic imbalances were called using binocular v0.2 (http://binocular .sourceforge.net). Binocular uses read coverage and variant allele frequencies to build likelihood that is used to segment chromosomes using a variant of the Circular Binary Segmentation algorithm. A Phred-scaled genotype likelihood (PL) cutoff ≥ 90 was used to define heterozygous germline loci. Segments were identified requiring a log likelihood ratio > 90, and that either ratio between copy numbers was > 1.1, or that difference in minor allele frequency was > 0.05 between the two segments. A p-value threshold of 0.001 was used to define segments with allelic imbalance. Copy numbers were normalized by multiplying them with a factor such that after normalization the 20^th^ percentile (weighted with respect to segment size) across segments with allelic balance was 2.

To infer the fraction of mutations on chromosome 8q that occurred before the arm was duplicated, we built a mixed model allowing for different fractions, copy numbers, *cn*, and tumor content, *tc*. For the fraction of mutations occurring before the duplication, number of variant reads was modeled as a binomial with mean *tc/cn*. For mutations occurring prior to the duplication half are not duplicated and follow the same binomial distribution as above, and for the remaining half the number of variant reads follows a binomial distribution with mean tc*(1 – 1/cn). Using this model and maximum likelihood we estimated fractions of mutations occurring before the chromosomal duplication.

### Analysis of mutational signatures

To estimate mutational signatures, we used a modified version of the methodology developed by Alexandrov and colleagues [20]. Given that this method optimally requires a larger number of samples than we have sequenced here, we modified it to use the 30 signatures that have been previously inferred from > 12,000 samples. We approximated the number of mutations of each signature type as *M* ≈ P × E, where P is the probability distribution for each signature and E describes the sample's exposure of each signature. We used the signatures available from COSMIC, and found the E that minimized the mean squared error with the condition that all elements are non-negative. To estimate the confidence interval of the estimated exposure the procedure above was repeated with added noise. Rather than using the number of observed mutations, Mij, we used a Poisson distributed random number with mean M_ij_. This was repeated 1,000 times, and the range from the 5^th^ to 95^th^ percentile defined the confidence interval.

### Sanger sequencing

Sanger sequencing was used to confirm *BAP1*, *SF3B1* and *EIF1AX* variants found by WGS/WES. Primers are listed in Supplementary Table 6. Sequencing reactions were performed using Big Dye V3.1 and run on an ABI 3730xl (Applied Biosystems). Sequencing results were analyzed using the chromatogram viewer, Chromas (version 1.45; Technelysium Pty Ltd).

## SUPPLEMENTARY MATERIAL FIGURES AND TABLES







## NOTE ADDED IN PROOF

While this manuscript was under review, the TCGA UM data were released (11/14/2015), which identified 2/80 samples with mutations in codon p.D630 of PLCB4. Sample TCGA-VD-A8KD carries two mutations at adjacent bases in the same codon, c.G1888T and c.A1889T, which if they are biallelic result in amino acid changes p.D630Y (the same mutation we describe here) and p.D630V, respectively, or if they occur on the same allele translate to p.D630F. Sample TCGA-YZ-A985 carries a c.G1888A mutation resulting in amino acid change p.D630N.
