# Supplementary material for: Deep sequencing of uveal melanoma identifies a recurrent mutation in PLCB4
Source: Oncotarget. 2015 Dec 14;7(4):4624–31. doi: 10.18632/oncotarget.6614 (PMC4826231; doi:10.18632/oncotarget.6614)
Supplement: Supplementary file 1 [file oncotarget-07-4624-s001.pdf]

SUPPLEMENTARY MATERIAL

SUPPLEMENTARY REFERENCE

1. Szklarczyk D, Franceschini A, Kuhn M, Simonovic M, Roth A, Minguez P, Doerks T, Stark M, Muller J, Bork P, Jensen LJ, von Mering C. The STRING database in 2011: functional interaction networks of proteins, globally integrated and scored. Nucleic acids research. 2011; 39(Database issue):D561–568.

Species	Protein		
Human	V	D	S
Chimp	V	D	S
Rhesus	V	D	S
Marmoset	V	D	S
Squirrel	V	D	S
Mouse	V	D	S
Rat	V	D	S
Guinea Pig	V	D	S
Rabbit	V	D	S
Pig	V	D	S
Sheep	V	D	S
Horse	V	D	S
Dog	V	D	S
Megabat	V	D	S
Elephant	V	D	S
Opossum	V	D	S
Platypus	V	D	S
Zebra Finch	V	D	S
Chicken	V	D	S
Lizard	V	D	S
X Tropicalis	V	D	S
Tetraodon	V	D	S
Zebrafish	V	D	S
Lamprey	V	D	S

Supplementary Figure S1: PLCB4 p.D630Y conservation across species.

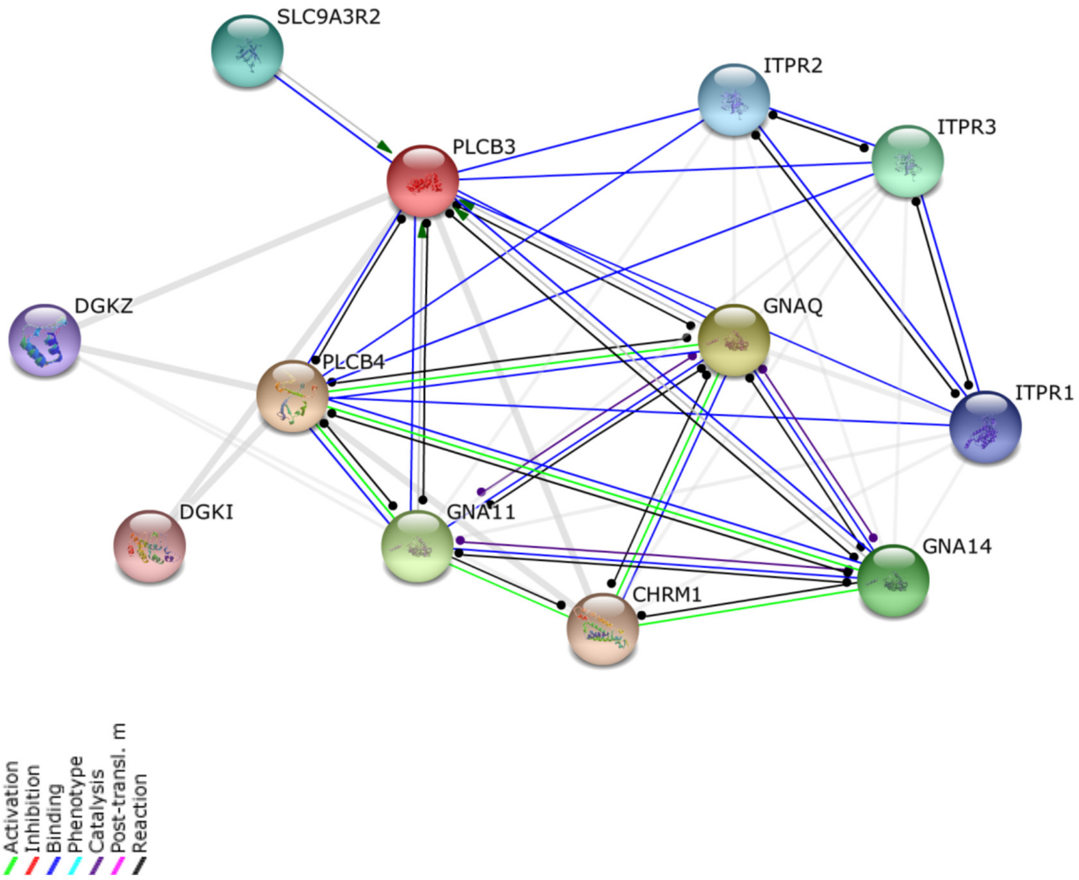

Supplementary Figure S2: PLCB4 interaction network generated by STRING [1].

Supplementary Table S1: Mutation burden per sample

Sample ID	Number of protein altering mutations	Exonic Mutations per Mb	Whole Genome Mutations per Mb^
MM1488-T	0	0.089	na
MM1563-T	0	0.059	na
554	0	0.059	0.22
K0111890AC-T	3	0.32	na
537	4	0.47	na
C0622943-T	5	0.26	na
MM1551-T	5	0.27	na
553	6	0.48	0.49
141378RG-T	6	0.39	na
538	7	0.36	0.35
534	8	0.53	0.63
533	8	0.48	0.55
ETB-0002-T	8	0.27	na
535	9	0.53	0.57
557	10	0.53	0.48
00038-001-CL	11	0.50	na
531	11	0.48	0.66
258	11	0.71	0.66
552	12	0.56	0.46
00061-001-CL	13	0.53	na
00085-001-CL	13	0.48	na
556	13	0.50	0.57
539	13	0.48	0.37
J2217960PR-T	14	0.65	na
00099-001-CL	15	0.59	na
550	19	0.83	0.60
MM639-T	19	0.80	na
00028-001-CL	53	2.52	na
**Average**	**10.6**	**0.52**	**0.50**

na is not applicable.
^ Mutations per Mb apply to whole-genome samples only.

Supplementary Table S2:

See Supplementary Table S2

Supplementary Table S3: Number of structural variants per sample

Sample ID	Number of structural variants	Chromosome 3
535	9	
552	11	LOH
537	12	
554	12	
533	15	LOH
534	16	LOH
538	16	LOH
557	17	
531	17	
258	21	LOH
553	24	LOH
539	27	copy neutral LOH
550	34	LOH
556	42	LOH
**TOTAL**	**273**	

LOH is loss of heterozygosity

Supplementary Table S4:

See Supplementary Table S4

Supplementary Table S5: Clinical details and histology of samples sequenced

Tumor ID	Specimen Type	Primary tumor site	Origin	Sex	Age at sample collection	Date of treatment	treatment	Cell type	Last Follow-up Date	Last Follow-up Status
00028-001-CL	primary cell line	left eye choroid	Australia	F	65	24/01/2012	enucleation	spindle type A (40%) and spindle type B (60%)	20/03/2015	na
00038-001-CL	primary cell line	left eye choroid	Australia	M	73	25/06/2012	enucleation	spindle type B, small component of epitheliod cells	01/07/2015	na
00061-001-CL	primary cell line	left eye choroid	Australia	M	70	7/08/2012	enucleation	spindle type B	13/07/2015	na
00085-001-CL	primary cell line	left eye choroid	Australia	F	63	15/10/2012	enucleation	spindle type B (50%) and epitheliod cells (50%)	01/12/2013	liver metastasis died 01/12/2013
00099-001-CL	primary cell line	right eye choroid	Australia	F	65	12/11/2012	enucleation	spindle type A (75%) and spindle type B (25%)	01/06/2015	na
C0622943-T	primary	choroid	Australia	M	64	20/12/2012	enucleation	mixed spindle and epithelioid	23/06/2015	na
141378RG-T	primary	cilary body	Australia	M	64	10/08/2012	enucleation	mixed spindle and epithelioid (predominantly spindle B)	23/06/2015	na
ETB-0002-T	primary	choroid	Australia	F	47	7/06/2011	enucleation	mixed spindle and epithelioid	23/06/2015	perineural spread
J2217960PR-T	primary	choroid	Australia	F	80	11/10/2012	enucleation	mixed spindle and epithelioid (predominantly spindle)	23/06/2015	na
K0111890AC-T	primary	choroid	Australia	M	85	20/04/2012	plaque/ enucleation	mixed spindle and epithelioid	23/06/2015	na
553	primary	choroid	Denmark	M	65	28/05/2014	plaque/ TVRC-biopsy	mixed spindle and epithelioid	21/10/2014	multiple liver metastasis
535	primary	choroid	Denmark	M	54	26/02/2014	plaque/ TVRC-biopsy	mixed spindle and epithelioid	26/02/2015	1.2 mm tumor
556	primary	choroid	Denmark	F	74	1/07/2014	plaque/ TVRC-biopsy	mixed spindle and epithelioid	8/01/2015	2.2 mm tumor
552	primary	choroid	Denmark	F	59	28/05/2014	enucleation	mixed spindle and epithelioid/ and invasion in sclerae	27/11/2014	progression free

(*continued*)

Tumor ID	Specimen Type	Primary tumor site	Origin	Sex	Age at sample collection	Date of treatment	treatment	Cell type	Last Follow-up Date	Last Follow-up Status
534	primary	choroid	Denmark	F	54	19/02/2014	plaque/ TVRC-biopsy	mixed spindle and epithelioid	13/03/2015	liver metastasis
533	primary	choroid	Denmark	F	73	5/02/2014	enucleation	mixed spindle and epithelioid/ and invasion in sclerae and extrascleral invasion	13/02/2015	progression free
557	primary	choroid	Denmark	M	61	29/07/2014	plaque/ TVRC-biopsy	spindle type	18/02/2015	1.5mm tumor
537	primary	choroid	Denmark	F	70	5/03/2014	plaque/ TVRC-biopsy	spindle type	12/09/2014	progression free
538	primary	choroid	Denmark	F	70	26/03/2014	plaque/ TVRC-biopsy	spindle type B, pre-existing nevus	18/09/2014	1.7mm tumor
531	primary	choroid	Denmark	F	63	8/01/2014	plaque/ TVRC-biopsy	mixed spindle and epithelioid	1/12/2015	0.9mm tumor
258	primary	choroid	Denmark	M	57	20/11/2013	plaque/ TVRC-biopsy	spindle type	31/07/2014	multiple liver metastasis
550	primary	choroid	Denmark	F	62	23/04/2014	enucleation	mixed spindle and epithelioid/ and invasion in sclerae	23/10/2014	progression free
554	primary	choroid	Denmark	F	39	11/06/2014	plaque/ TVRC-biopsy	spindle type	9/02/2015	1.2mm tumor
539	primary	choroid	Denmark	M	63	9/04/2014	plaque/ TVRC-biopsy	spindle type B	16/10/2014	2.8mm tumor
MM1488	liver metastasis	na	Sweden	M	36	2008	na	na	15/01/2015	na
MM1551	na	na	Sweden	na	na	na	na	na	na	na
MM1563	liver metastasis	na	Sweden	F	69	2008	na	na	15/02/2015	na
MM639	iliac crest metastasis	choroid	Sweden	M	79	15/12/1989	na	mixed spindle and epithelioid	19/11/1999	died 19/11/1999

TVRC-biopsy is transvitreal retino-choroidal biopsy

Supplementary Table S6: Primers used for Sanger sequencing verification

Primer	Sequence
BAP1 exon 3 forward	atgaataagggctggctggag
BAP1 exon 3 reverse	gctgctgctttctgtgagatt
BAP1 exon 4 forward	tcagaggcttatgctttgctc
BAP1 exon 4 reverse	tcctcctgtcttcctccattt
BAP1 exon 6 and 7 forward	tccacccatagtcctacctga
BAP1 exon 6 and 7 reverse	gggcaatatggtgtagggtga
BAP1 exon 9 forward	ttccagataggcccctcatac
BAP1 exon 9 reverse	gtggttagctgaagcccagat
BAP1 exon 11 forward	ggggagactgtgagcttttct
BAP1 exon 11 reverse	atcaggcagaggaacctagca
BAP1 exon 14 forward	cgtgttgactgcatacgctac
BAP1 exon 14 reverse	ccaatcttcacaccaaagttcc
BAP1 exon 16 forward	ccttgcctctagctgcctatt
BAP1 exon 16 reverse	caaggtctgctcaagcctca
BAP1 exon 17 forward	ctgaggcttgagcagacctt
BAP1 exon 17 reverse	acctggcttccttacaacagg
EIF1AX p.P2L forward	gaaaagcgacgcaaagagtc
EIF1AX p.P2L reverse	caaactgggcaacaggagtt
EIF1AX p.G6V, p.G8R and splice forward	tccaagaagggtagggaggt
EIF1AX p.G6V, p.G8R and splice reverse	tggtggtgtgtgcctgtagt
SF3B1 p. R625 and p.K666T forward	tagagtggaaggccgagaga
SF3B1 p.R625 and p.K666T reverse	gctgagcaggaggatcactt
